# A Novel Real-Time RT-PCR-Based Methodology for the Preliminary Typing of SARS-CoV-2 Variants, Employing Non-Extendable LNA Oligonucleotides and Three Signature Mutations at the Spike Protein Receptor-Binding Domain

**DOI:** 10.3390/life11101015

**Published:** 2021-09-27

**Authors:** Serafeim C. Chaintoutis, Taxiarchis Chassalevris, Sofia Balaska, Evangelia Mouchtaropoulou, George Tsiolas, Ioannis Vlatakis, Areti Tychala, Dimitris Koutsioulis, Anagnostis Argiriou, Lemonia Skoura, Chrysostomos I. Dovas

**Affiliations:** 1 Diagnostic Laboratory, School of Veterinary Medicine, Faculty of Health Sciences, Aristotle University of Thessaloniki, 11 S. Voutyra Str., 54627 Thessaloniki, Greece; schainto@vet.auth.gr (S.C.C.); taxiarchis@vet.auth.gr (T.C.); 2 Department of Microbiology, AHEPA University Hospital, Medical School, Faculty of Health Sciences, Aristotle University of Thessaloniki, S. Kyriakidi Str., 54636 Thessaloniki, Greece; sofiabalaska8417@gmail.com (S.B.); arettych@auth.gr (A.T.); lemskour@auth.gr (L.S.); 3 Institute of Applied Biosciences, Centre of Research and Technology Hellas, Thermi, 57001 Thessaloniki, Greece; eva.mouchtaropoulou@certh.gr (E.M.); george.tsiolas@certh.gr (G.T.); argiriou@certh.gr (A.A.); 4 EnzyQuest PC, Science and Technology Park of Crete, 100 N. Plastira Str., Vassilika Vouton, 70013 Heraklion, Greece; vlatakis@enzyquest.com (I.V.); koutsioulis@enzyquest.com (D.K.); 5 Department of Food Science and Nutrition, University of the Aegean, 81400 Myrina, Greece

**Keywords:** SARS-CoV-2, real-time RT-PCR, typing, spike protein, receptor-binding domain, variant of concern, mutation, LNA oligonucleotide blockers

## Abstract

Mutations resulting in amino-acid substitutions of the SARS-CoV-2 spike protein receptor-binding domain (RBD) have been associated with enhanced transmissibility and immune escape of the respective variants, namely Alpha, Beta, Gamma or Delta. Rapid identification of the aforementioned variants of concern and their discrimination of other variants is thus of importance for public health interventions. For this reason, a one-step real-time RT-PCR assay employing four locked nucleic acid (LNA) modified TaqMan probes was developed, to target signature mutations associated with amino-acid substitutions at positions 478, 484 and 501 present in the receptor-binding motif (RBM) of the spike protein RBD. This region contains most contacting residues of SARS-CoV-2 that bind to ACE2. A novel strategy employing the use of non-extendable LNA oligonucleotide blockers that can reduce non-specific hybridization of probes increased the number of different mutated sites examined in a multiplex PCR. The combinatory analysis of the different fluorescence signals obtained enabled the preliminary differentiation of SARS-CoV-2 variants of concern. The assay is sensitive with a LOD of 263 copies/reaction for the Delta variant, 170 copies/reaction for the Beta variant, amplification efficiencies > 91% and a linear range of >5 log_10_ copies/reaction against all targets. Validation of the assay using known SARS-CoV-2-positive and negative samples from humans and animals revealed its ability to correctly identify the targeted mutations and preliminary characterize the SARS-CoV-2 variants. The novel approach for mutation typing using LNA oligonucleotide blockers can be modified to target signature mutations at four different sites in the RBM and further expand the range of variants detected.

## 1. Introduction

The continuous emergence of mutations resulting to amino-acid substitutions in the spike (S) protein of severe acute respiratory syndrome coronavirus 2 (SARS-CoV-2) is of major importance. This is due to the fact that some of them are associated with alterations in host cell receptor binding leading to increased infectivity and transmissibility, as well as in poor antigen/antibody interactions, reduced neutralization and consequently, immune escape [1]. The S:D614G (aspartic acid to glycine) amino-acid substitution emerged early in the SARS-CoV-2 evolutionary history and has been associated with higher virus infectivity [2]. A variant possessing this substitution became rapidly dominant throughout the world, and further evolved to give several variants of concern (VOCs) [3]. Thus, the aforementioned substitution is now ubiquitous worldwide. Until recently, SARS-CoV-2 VOCs with recognized importance, according to the CDC, included the Alpha variant (B.1.1.7 lineage), Beta variant (B.1.371 lineage) and Gamma variant (P.1 lineage). Among these 3 VOCs the S:N501Y (asparagine to tyrosine) is a shared amino-acid substitution in the receptor-binding domain (RBD) [4]. The S:E484K amino-acid substitution (glutamate to lysine) is also shared between the Beta and Gamma variant strains [5]. The Delta variant (lineage B.1.617.2) is a VOC that emerged more recently. A novel S:T478K (threonine to lysine) substitution in the RBD is so far a unique characteristic of Delta variant strains [6], that since January 2021 has been found in increasing frequencies in several countries [7]. This variant is also characterized by decreased sensitivity to antibody-mediated neutralization [8], thus immediate identification for its presence in infected individuals is critical, as is for the three aforementioned VOCs. Beyond the described VOCs, other SARS-CoV-2 variants, which have been designated as variants under monitoring or variants under investigation are also of importance. Lineages categorized within such variants also possess some of the described mutations and amino-acid substitutions, e.g., the 484K phenotype is present in lineage B.1.1.318, which is categorized under the Iota variant [9,10]. As a result, differentiation among VOCs and other variants is deemed necessary.

Despite the fact that the next-generation sequencing (NGS)-based SARS-CoV-2 genome analysis can characterize a given variant with the highest accuracy, its application is time-consuming, cost-inefficient, labor-intensive and not readily available in the majority of the diagnostic laboratories. On the other hand, PCR-based assays for variant identification or typing of mutations related to specific amino-acid substitutions are rapid, performed simply and are applicable in many diagnostic laboratories. The use of such assays for the early detection and determination of the prevalence of selected variants has also been suggested by international organizations [11]. Our research team has recently developed a sensitive and reliable real-time RT-PCR assay which can be used as a fast screening tool to preliminary differentiate variants containing mutations of concern in two sites of the RBD [12]. Aim of this study was to develop a novel real-time RT-PCR-based methodology able to identify signature mutations present in more than two sites of the viral receptor-binding motif (RBM) that contains most of the contacting residues of SARS-CoV-2 that bind to ACE2. This was feasible by employing non-extendable locked nucleic acid (LNA) oligonucleotides that reduce non-specific hybridization of probes. Based on the combinatory interpretation of the obtained fluorescence signals, the developed assay enables the preliminary characterization of VOCs and other SARS-CoV-2 variants under investigation or monitoring with potential public health impact.

## 2. Materials and Methods

### 2.1. Primers, TaqMan Probes and Non-Extendable Blocking Oligonucleotides

Two primers (DSarsUp1 and SARSpDo1; Table 1) were designed, so as to amplify a 291 bp amplicon of SARS-CoV-2 S gene (positions 22,843–23,133 on GenBank acc. No. NC_045512), flanking the RBM-coding region for amino-acids S438-515. Both primers were evaluated in silico with PrimerChecker tool offered by GISAID [13]. Primers were compared against 1,257,634 high quality SARS-CoV-2 genomes submitted, spanning between 14 January 2021 and 15 July 2021. Tables derived from GISAID were transformed with dplyr package in R Studio and the results were visualized with Microsoft Office Excel, in order to assess the nucleotide substitutions found on primer hybridization regions. Further analysis was performed in silico using the DINAMelt software [14] to estimate, at the annealing temperature of 58 °C, the mole fraction of each primer hybridized to the target sequences with most common single mismatches in SARS-CoV-2 genomic sequences (frequency > 4%) [15].

Four LNA TaqMan probes were used, each conjugated with a different fluorophore at the 5’ end (FAM, HEX, Texas Red and Cy5) for fluorescence signal discrimination (Table 1). Selection of the aforementioned fluorophores to enable multiplexing on the qPCR instrument used was performed through the PrimeTime Multiplex Dye Selection Tool [16] offered by IDT (Integrated DNA Technologies, Coralville, IA, USA). Sequences from the 3 of the 4 probes were published previously [12]. More specifically, 2 of these probes target the mutation associated with the E484K amino-acid substitution, enabling differentiation of two phenotypes (FAM: 484E; Texas Red: 484K), and a third probe is used for the identification of the 501Y phenotype at the Cy5 fluorescence channel. An additional TaqMan probe was designed to enable identification of the mutation associated with the 478K phenotype, at the HEX fluorescence channel. The rationale and principles behind the design of the new TaqMan probe were the same as for the other 3 probes, and have been previously described [12].

For substitutions at positions 478 and 501, that are being targeted by only one TaqMan probe, two respective complementary non-extendable blocking oligonucleotides with LNA modifications to target phenotypes 478T and 501N were designed (Inh478Τ and Inh501Ν; Table 1) and included in the assay. This novel strategy reduced non-specific fluorescence signals in cases of strains without the targeted mutations, as the design of additional competing probes for wild type (WT) sequences was not possible due to the reduced capability for fluorophore multiplexing, which is usually 4. Both blockers were modified with a 3’ C3 spacer terminator to inhibit polymerase extension at their 3’ end [17]. The concentration of blockers was adjusted on the basis of preliminary RT-PCR runs, where the same samples were tested in multiple reactions, with and without blocking oligonucleotides. The final blocker concentration was chosen so as to significantly reduce non-specific fluorescence, without affecting the specific fluorescence signals in the developed assay. All primers and blockers were purchased from IDT (Integrated DNA Technologies, Coralville, IA, USA) at a purity grade of standard desalting.

### 2.2. Real-Time RT-PCR

The reaction mix composition and the thermal cycling conditions were optimized using EnzyQuest’s One-step RT-qPCR kit (Product No.: RN010; EnzyQuest P.C., Heraklion, Greece). In this case, 20-μL reactions were comprised by: 4 mM Mg^2+^, all 8 oligonucleotides described above, at the concentrations shown in Table 1, and 2 μL of sample RNA. The reactions were optimized on a CFX96 Touch Real-Time PCR Detection System (Bio-Rad Laboratories, Hercules, CA, USA). Fluorescence data acquisition and analysis were performed using the CFX Maestro Software (v4.1; Bio-Rad Laboratories, Hercules, CA, USA). Cycling conditions were as follows: 55 °C for 15 min (reverse transcription), 94 °C for 15 min (inactivation of reverse transcriptase and activation of Taq polymerase), and 48 cycles in steps: (a) 94 °C for 10 s (denaturation), (b) 58 °C for 40 s (annealing) and (c) 72 °C for 5 s (extension). Measurement of fluorescence levels was being performed at the end of step b of each cycle.

### 2.3. Analytical Characteristics

The analytical parameters of the developed assay were determined as follows: RNA extracts from two SARS-CoV-2-positive nasopharyngeal clinical samples, i.e., one Delta variant strain (fluorescence: FAM/HEX) and one Beta variant strain (fluorescence: Texas Red/Cy5) were quantified using CDC’s N2 real-time RT-PCR, as described previously [18]. Both quantified sample RNAs were serially (10-fold) diluted. A SARS-CoV-2-negative RNA extract originating from human oropharyngeal swabs was used as a dilution background (diluent). Dilutions representing 4 × 10^6^ down to 4 × 10^2^ copies/assay for Delta variant and 1.5 × 10^6^ down to 1.5 × 10^2^ for Beta variant were thus prepared and were subsequently tested to determine the linear range and amplification efficiency of the developed assay for both targeted VOCs, with each dilution being run in triplicate. Both aforementioned RNA extracts were further 2-fold diluted at 600, 300, 150, 75, 37.5 and 18.75 copies/reaction. The prepared dilutions were tested in 12 technical replicates each and the limit of detection (LOD) was determined with 95% probability of detection, using the Quodata web application [19]. The analytical characteristics were determined using a CFX96 Touch Real-Time PCR Detection System.

### 2.4. Specificity and Diagnostic Performance

To validate the diagnostic performance of the newly developed typing real-time RT-PCR assay in clinical specimens, two panels of SARS-CoV-2-positive RNA extracts originating from human nasopharyngeal samples (N = 101, Table 2) were tested with the assay described herein, on a CFX96 Touch Real-Time PCR Detection System (Bio-Rad Laboratories, Hercules, CA, USA).

Panel A of positive human samples (N = 60) originated from the AHEPA university hospital, one of the reference hospitals for COVID-19 in Northern Greece. These samples were obtained either for the laboratory diagnosis COVID-19, or for tracing close contacts. Samples were positive based on the application of either the Abbott RealTime SARS-CoV-2 assay on the m2000 RealTime System, or the NeuMoDx SARS-CoV-2 Assay on the NeuMoDx 96 Molecular System. In this case, 26 out of the 60 positive samples were collected between February and April 2021. The ViroBOAR Spike 1.0 RT-PCR Kit (Eurofins Genomics, Ebersberg, Germany) was used for their characterization, using a LightCycler 480 II instrument (Roche). Based on this testing, they were classified as follows: 4 WT strains, 21 Alpha variant strains and 1 Beta variant strain (Table 2). Samples characterized as Delta variant strains (14 out of 60) were also included. Their characterization was based on the use of the SNPsig SARS-CoV-2 (EscapePLEX) mutation detection/allelic discrimination Kit targeting the E484K, K417N/T and P681 substitutions (Primerdesign) on an AriaMx Real-Time PCR System (Agilent Technologies, Santa Clara, CA, USA). Additional human WT strains (20 out of 60), obtained during September 2020, prior to the emergence of the targeted mutations in Greece [20] were also included. RNA extraction from the aforementioned positive specimens was performed by means of the NucleoSpin RNA Virus kit (Macherey-Nagel, Düren, Germany).

Panel B of positive human samples (N = 41) originated from the National Flagship Action “Greece vs Corona”. The MagMAX Viral/Pathogen Nucleic Acid Isolation kit (ThermoFisher Scientific, Waltham, MA, USA) and the KingFisher Flex instrument (ThermoFisher Scientific, Waltham, MA, USA) were used for RNA extraction. Strains were characterized by NGS (Illumina MiSeq) as follows: 2 Alpha variant strains, 2 Beta variant strains, 36 Delta variant strains and one was a strain of the lineage B.1.1.318 (within the Iota variant), in which only the 484K phenotype is present out of those targeted by the assay (Table 2).

To assess the specificity of the developed assay a panel of RNA extracts from SARS-CoV-2-negative human nasopharyngeal swabs (N = 20) was also tested on a CFX96 Touch Real-Time PCR Detection System. The samples originated from the same hospital setting as was the panel A of positive human samples and screening was performed similarly.

A collection of animal samples was also tested via the developed assay, due to the fact that SARS-CoV-2 is of veterinary importance as well. RNA extracts from oropharyngeal swabs obtained from previously investigated cats (N = 2) infected by a WT strain (B.1.1 lineage) were analyzed [18]. SARS-CoV-2-positive oropharyngeal samples from minks (N = 3) infected by a WT strain (B.1.1.305 lineage) originating from a heavily affected farm were tested [21]. Six negative samples from cats (N = 3) and minks (N = 3) were tested for specificity purposes. Testing of the specimens comprising the veterinary panel was also performed on a CFX96 Touch Real-Time PCR Detection System (Bio-Rad Laboratories, Hercules, CA, USA).

## 3. Results

**Figure 1:**
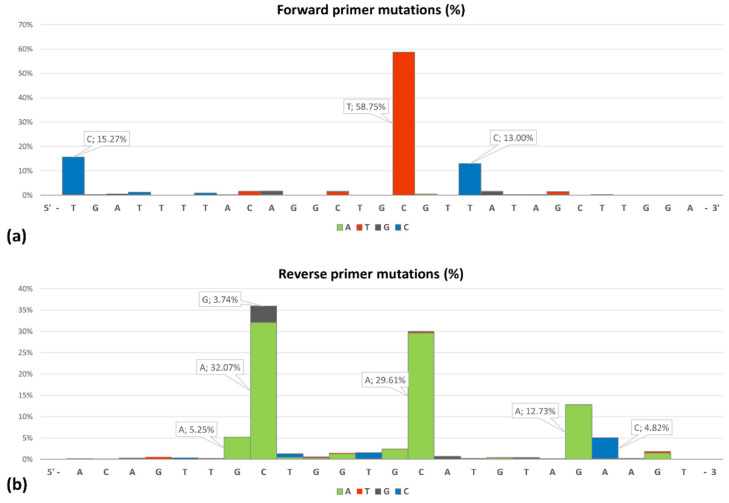
Single nucleotide mutation frequencies of SARS-CoV-2 homologous S gene sequences spanning between 14 January 2021 and 15 July 2021 as compared to the forward primer (**a**) and the reverse primer (**b**).

**Figure 2:**
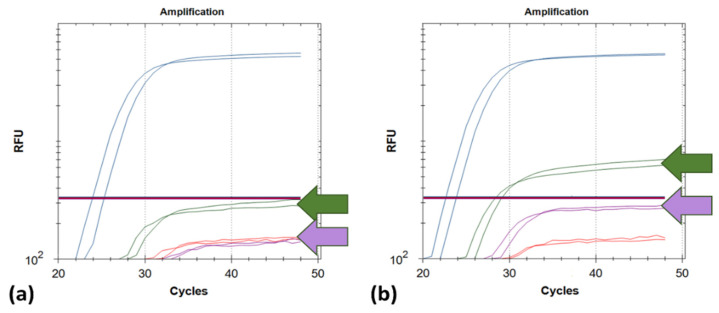
Comparison of the fluorescence signals in all four channels (FAM, HEX, Texas Red and Cy5) by testing 2 different wild type SARS-CoV-2-postive RNA extracts with the developed assay in the presence (**a**) and absence (**b**) of oligonucleotide blockers for positions 478 (HEX, green) and 501 (Cy5, violet). The specific fluorescence signal at the FAM channel (blue; 484E) was not affected.

Comparisons for primer DSarsUp1 revealed single nucleotide mismatches in 4254 genomic sequences (Figure 1) and 2 with triple and quadruple nucleotide mismatches, whereas for primer SARSpDo1, only single and double mismatches were found in 7654 and 19 genomic sequences, respectively. In silico estimation of equilibrium melting profiles of the mismatched duplexes revealed that all sequences with single mismatches (Figure 1) are expected to be amplified. More specifically, at 58 °C, the mole fractions of primer DSarsUp1 hybridized to the targeted variants with single mismatches, ranged from 78 to 98%, whereas those of SARSpDo1 ranged from 48 to 80%, respectively. Both LNA oligonucleotide blockers reduced non-specific fluorescence (55% for Hex and 49% for Cy5) without affecting the specific FAM fluorescence signals (Figure 2).

**Figure 3:**
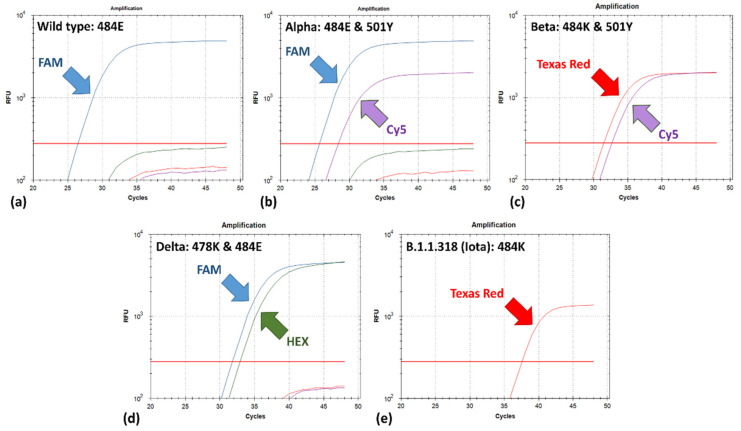
Fluorescence interpretation algorithm from testing representative SARS-CoV-2 variants (shown on the upper left corner), indicating possible S protein phenotypes based on the combination of fluorescence signals obtained, namely: 484E/WT phenotype (**a**), 484E and 501Y phenotype, such as the Alpha variant (**b**), 484K and 501Y phenotype, e.g., the Beta variant (**c**), 478K and 484E, such as the Delta variant (**d**) and strains with only the 484K phenotype, such as of the B.1.1.318 lineage which is now listed within the Iota variant (**e**).

Based on the combinatory analysis of the fluorescence signals obtained from the four channels, the type of the investigated SARS-CoV-2 strain can be preliminarily identified as: (i) a WT strain, i.e., a strain with none of the three targeted amino-acid substitutions, wherein fluorescence is obtained only in the FAM channel (indicative of the 484E phenotype), whereas no fluorescence is acquired for the other two positions due to the inhibition; (ii) a strain with the 484E (FAM) and 501Y (Cy5) phenotype, such as an Alpha variant strain; (iii) a strain with the 484K (Texas Red) and 501Y (Cy5) phenotype, such as a Beta or a Gamma variant strain; (iv) a strain with a 478K (HEX) and 484E (FAM) phenotype, such as a Delta variant strain, as well as v) viral strains or variants characterized only by the E484K (HEX) substitution, out of those targeted, such as of the B.1.1318 lineage (Figure 3).

**Figure 4:**
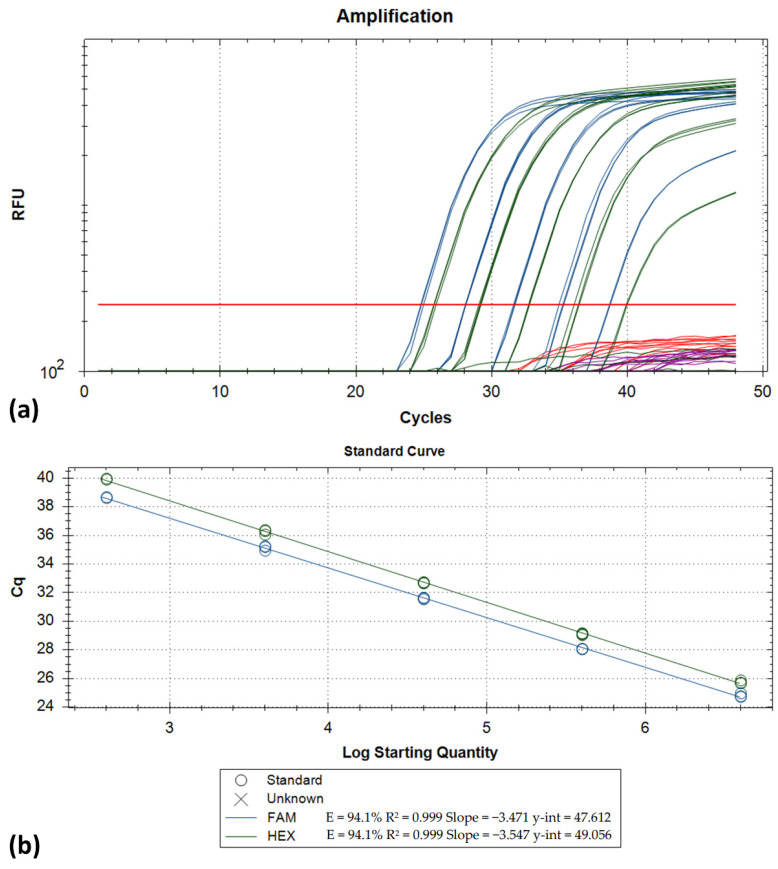
(**a**) Amplification plots generated by testing a dilution series (10-fold dilutions 4 × 10^6^ down to 4 × 10^2^ copies/assay from left to right) of a Delta variant strain RNA extract (FAM and HEX fluorescence channels). Each dilution was run in triplicate; (**b**) the corresponding standard curves.

**Figure 5:**
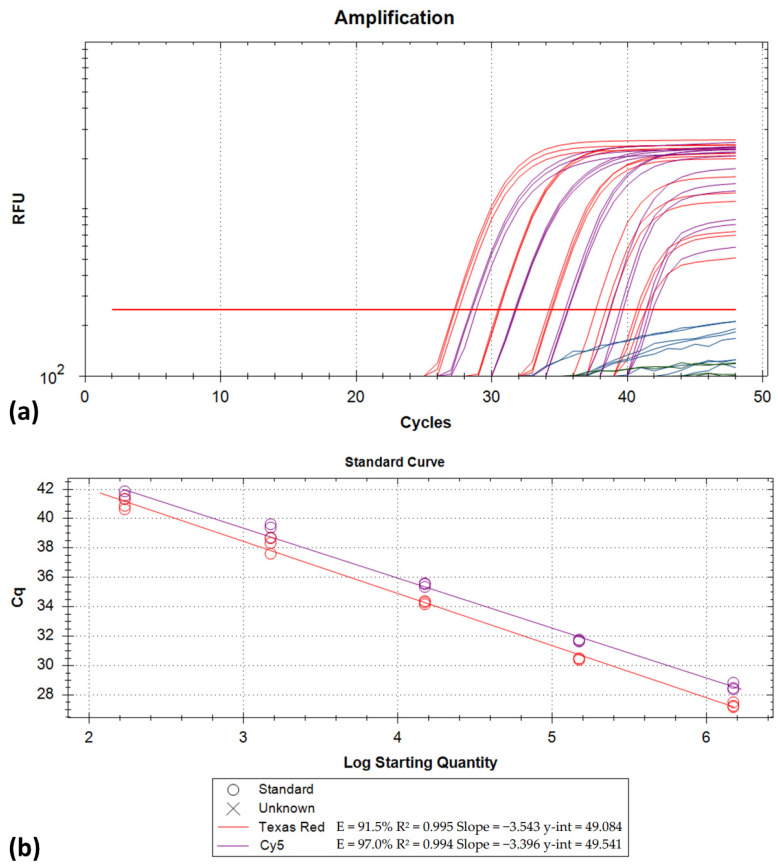
(**a**) Amplification plots generated by testing a dilution series (10-fold dilutions 1.5 × 10^6^ down to 1.5 × 10^2^ copies/assay from left to right) of a Beta variant strain RNA extract (Texas Red and Cy5 channels). Each dilution was run in triplicate; (**b**) the corresponding standard curves.

The amplification efficiency for both the Delta and the Beta variant strains was determined to be > 91%. Specifically, for the Delta variant, efficiencies were 94.1% for FAM and 91.4% for HEX (Figure 4). Similarly, the amplification efficiencies for the Beta variant strain were determined at 91.5% for Texas Red and 97.0% for Cy5. For both variants, a linear range of quantification > 5 log_10_ was observed (Figure 5). The LOD for the developed assay was 263 copies/reaction for the Delta variant (FAM/HEX; 95%CI: 179.4–387.5) and 170 copies/reaction for the Beta variant (Texas Red/Cy5; 95%CI: 115.1–252.9).

All tested variants in the 106 SARS-CoV-2-positive samples from humans and animals were correctly identified and discriminated, verifying the validity of the developed assay regarding the identification of the targeted mutations (Table 2). Testing of the 26 SARS-CoV-2-negative RNA extracts of human and animal origin revealed the absence of fluorescence in all four channels, confirming the specificity of the assay.

## 4. Discussion

Diversification within the RBD of SARS-CoV-2 is driven by host adaptation, due to positive selection and putative epistatic interactions [22]. The RBD has a receptor-binding motif (amino-acids 438–506) that contains most of the contacting residues of SARS-CoV-2 that bind to ACE2 and regulate the infectivity, pathogenesis, and cross-species and human-to-human transmission of SARS-CoV-2 [23,24]. Amino-acid substitutions within this region result in significant phenotype changes, i.e., enhance binding to the ACE2 receptor [4] and/or reduce binding of polyclonal convalescent plasma antibodies [25], and therefore, their combination characterizes emerging variants. In the present work, we amplify the RBM-coding region and by using a novel approach, we target signature mutations in multiple sites using real-time RT-PCR for initial screening of VOCs and other variants of interest (VOIs). Based on the combinatory analysis of the fluorescence signals obtained, mutant SARS-CoV-2 strains can be preliminarily typed into variants, such as the Alpha, Beta/Gamma and Delta VOCs, or grouped among other variants with increasing interest for public health. Strains without mutations in the targeted positions (characterized as wild type) can also be identified. The real-time RT-PCR-based format of the assay enables its widespread applicability, rapid turnaround and reduced cost for SARS-CoV-2 variant identification. Its application is thus expected to restrict the need for NGS-based analyses to only selected samples, for which the assay presented herein will constitute a rapid pre-screening tool.

Despite the fact that the methodology described is based on real-time PCR, a novel approach for discrimination of targeted mutations in more than two genomic sites was developed, based on the use of different LNA-modified oligonucleotides. LNA modifications provide sufficient mismatch discrimination by increasing the stability of LNA·DNA duplexes and allow the use of shorter probes in which single mismatches have a large impact on the duplex stability, especially when mismatch sites are located in the center of the duplex. In addition, triplets of LNA residues containing the mismatch in the center can increase specificity directly since mismatches in LNA·DNA duplexes are more destabilizing than in the case of DNA·DNA duplexes [26]. Usually, PCR annealing temperatures 3−5 °C below the *Tm* of the probes allow stability of perfectly matched probe−target duplexes, while under these conditions the mismatched duplexes are likely to be unstable and will not give a false positive fluorescence signal. Base pair nearest-neighbor parameters affect mismatch discrimination of LNAs and specificity is dependent on mismatch type and adjacent bases. However, there are cases of sequences where level of mismatch discrimination decreases and non-specific hybridization on mismatched mutants can occur [26]. In these cases, a matching probe can effectively compete the hybridization of a homologous probe with mismatch and reduce non-specific fluorescence signal for the same DNA target. However, this approach reduces the number of mutated sites that can be tested in real-time PCR instruments. In this paper we propose the use of non-extendable oligonucleotide blockers that can reduce non-specific hybridization of probes and increase the number of different mutated sites that can be tested in a multiplex PCR.

In conclusion, through the design and use of modified oligonucleotides, sufficient mismatch discrimination with high sensitivity is supported. The novel real-time RT-PCR which is presented herein enables the preliminary characterization of SARS-CoV-2 VOCs, as well as for the investigation of other VOIs in human and animal specimens. In the possible future emergence of strains in which the 478K and the 484K will both be present, the assay is expected to also be able to identify them correctly and discriminate them from the others. Further modifications of this assay are also feasible, via the incorporation of an additional TaqMan probe and a homologous non-extendable oligonucleotide blocker targeting a fourth signature mutation of the RBD-coding region amplified, e.g., the mutation associated with the L452R amino-acid substitution, which is also a shared mutation between the Delta, Epsilon and Kappa variants, or to identify the Lambda variant, that instead of the L452R possesses the L452Q substitution [6]. This improvement will enable a more precise variant identification, via a more complex fluorescence interpretation algorithm, highlighting further the usefulness of the assay as a rapid tool for screening of SARS-CoV-2 variants and calculation of their prevalence in the context of epidemiological studies. The developed assay can only serve as a mutation identification tool in SARS-CoV-2-positive specimens, thus is not aimed to be used as a routine screening assay, despite its high sensitivity. This is due to the fact that additional mutations which could emerge in the probe-binding regions might prevent their hybridization. Additional amino-acid substitutions located outside the RBM and affecting ACE2 receptor binding could emerge in the future. Given that the genomic region targeted by the assay presented herein cannot be expanded without compromising the amplification efficiency and LOD, adaptation of the protocol would be required via the design of novel primers, probes and blocking oligonucleotides, based on the principles described herein. Lastly, the proposed molecular typing methodology can be expanded to prokaryotic and eukaryotic organisms, for the detection of SNPs in more than two positions.

## Figures and Tables

**Table 1:** Primers, TaqMan probes and non-extendable oligonucleotide blockers of the developed assay.

Oligonucleotide Name (Fluorophore)	Sequence (5′–3′)	Hybridization on Acc. NC_045512	*Tm* (°C)	Conc. (μΜ)
DSarsUp1	TGATTTTACAGGCTGCGTTATAGCTTGGA	22,843–22,871	69.0	0.20
SARSpDo1	ACAGTTGCTGGTGCATGTAGAAGT	23,110–23,133	66.5	0.20
Probe 478K (HEX)	ACA+AGG+T+**T**+TGCT+ACC	22,910–22,924	63.1	0.40
Probe 484Εw (FAM) *	AA+AACCT+T+**C**+AACACCA	23,005–23,019	62.9	0.12
Probe 484Κ (Tex.Red) *	AA+AACCT+T+**T**+AA+CACCA		62.2	0.07
Probe 501Y (Cy5) *	AACACCA+T+**A**+AGTGGGT	23,056–23,071	62.7	0.30
Inh478T	ACA+AGGT**G**TGCTA+CC/3SpC3/	22,910–22,924	61.5	0.50
Inh501N	ACACCA+T+**T**+AGTGGGT/3SpC3/	23,057–23,071	62.7	0.30

+: Locked Nucleic Acid (LNA) modifications; *: previously published sequences; 3SpC3: 3’-end C3 Spacer terminator. Mismatch positions are indicated in boldface.

**Table 2:** Panels of SARS-CoV-2 positive (N = 106) and negative (N = 26) human and veterinary samples tested for validation of the developed assay.

Panel	Mutation Identification Method	No.	Variant	Fluorescence			
				F	H	T	C
**Negative**	**N/A**	**20**	N/A	-	-	-	-
**Positive (A)**	Obtained prior to the emergence of the target mutations	20	WT	+	-	-	-
	ViroBOAR Spike 1.0 RT-PCR Kit	4	WT	+	-	-	-
		21	Alpha	+	-	-	+
		1	Beta	-	-	+	+
	SNPsig SARS-CoV-2 (EscapePLEX)	14	Delta	+	+	-	-
**Positive (B)**	NGS (Illumina MiSeq)	2	Alpha	+	-	-	+
		2	Beta	-	-	+	+
		36	Delta	+	+	-	-
		1	B.1.1.318	-	-	+	-
**Negative (Vet)**	N/A	3 cats, 3 minks	N/A	-	-	-	-
**Positive (Vet)**	NGS (Ion Torrent GeneStudio S5)	2 cats	WT	+	-	-	-
	NGS (Illumina MiSeq)	3 minks	WT	+	-	-	-

+: fluorescence detection in the respective channel; -: absence of fluorescence in the respective channel; F: FAM; H: HEX; T: Texas Red; C: Cy5; N/A: not applicable; NGS: next-generation sequencing; WT: wild type.

## Data Availability

Not applicable.
